# Characterization of the structure and expression of mouse *Itpa* gene and its related sequences in the mouse genome

**DOI:** 10.1093/dnares/dsv006

**Published:** 2015-04-17

**Authors:** M. Behmanesh, K. Sakumi, D. Tsuchimoto, K. Torisu, Y. Ohnishi-Honda, D. E. Rancourt, Y. Nakabeppu

*DNA Research*, 2005, 12(1), 39–51; doi: 10.1093/dnares/12.1.39

The authors did not fully acknowledge the original publication when they reused part of a figure in their Fig. 7B in the above article. The legend for Figure 7 should read as follows. A new version of Fig. 7C is also given below as the labels on the *x*- and *y*-axes were not visible in the original published version. The authors would like to apologize for these errors.

**Figure 7.**
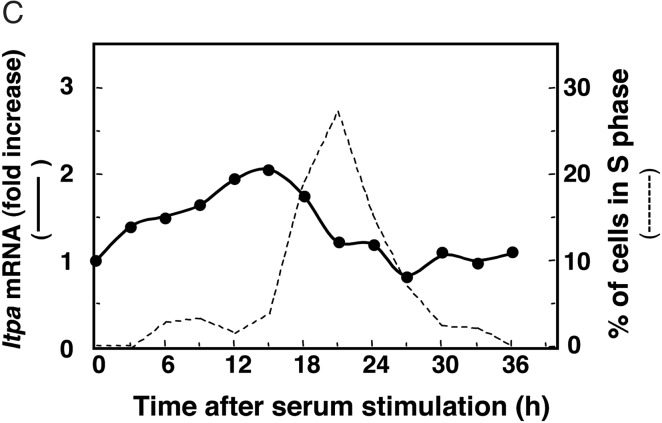
Expression of mouse *Itpa* gene in mouse tissue and its association with cell proliferation. *A*. The expression of *Itpa* mRNA in various types of adult mouse tissue. Total RNAs (20 μg each) extracted from various types of mouse tissue were electrophoresed, transferred onto a Hybond^TM^-N^+^ nylon membrane, and subsequently probed with ^32^P-labeled fragment containing the entire coding region from the type A *Itpa* cDNA (731 bp) (top panel) and the 18S rRNA probe^49^ (middle panel), as previously described.^27,50^ The arrowhead indicates *Itpa* mRNA. In the bottom panel, the relative amounts of *Itpa* mRNA to 18S rRNA were calculated based on the radioactivity. The ratio of the relative amount of each transcript to that in the testis is shown. *B*. The expression of *Itpa* mRNA in quiescent and serum-stimulated BALB/c3T3 cells. The total RNA isolated was subjected to Northern blot analyses to determine the expression amount of *Itpa* mRNA and 18S rRNA. The data for 18S rRNA was originally published in Ide Y, Tsuchimoto D, Tominaga Y, Iwamoto Y, Nakabeppu Y, *Genomics*, **81**, 47–57, 2003^27^, **©** Elsevier**,** and the same blot was used to detect *Itpa* mRNA in the present study. *C*. The relative amounts of *Itpa* mRNA to 18S rRNA were calculated based on radioactivity. The ratio of the relative amount of each transcript to that in the quiescent cells is shown (closed circle). The percentages of the cells in the S phase of the cell cycle were determined by flow cytometry as previously described^27,28^ and then were plotted with a dotted line.

